# Widening the infantile hypotonia with psychomotor retardation and characteristic Facies-1 Syndrome’s clinical and molecular spectrum through NALCN *in-silico* structural analysis

**DOI:** 10.3389/fgene.2024.1477940

**Published:** 2024-12-11

**Authors:** Davide Vecchio, Marina Macchiaiolo, Michaela V. Gonfiantini, Filippo M. Panfili, Francesco Petrizzelli, Niccolò Liorni, Fabiana Cortellessa, Lorenzo Sinibaldi, Ippolita Rana, Emanuele Agolini, Dario Cocciadiferro, Nicole Colantoni, Michela Semeraro, Cristiano Rizzo, Annalisa Deodati, Nicola Cotugno, Serena Caggiano, Elisabetta Verrillo, Carlotta G. Nucci, Serpil Alkan, Jorge M. Saraiva, Joaquim De Sá, Pedro M. Almeida, Jayanth Krishna, Paola S. Buonuomo, Diego Martinelli, Carlo Dionisi Vici, Viviana Caputo, Andrea Bartuli, Antonio Novelli, Tommaso Mazza

**Affiliations:** ^1^ Rare Diseases and Medical Genetics Unit, Bambino Gesù Children’s Hospital, IRCCS, Rome, Italy; ^2^ Bioinformatics Unit, Fondazione IRCCS Casa Sollievo Della Sofferenza, San Giovanni Rotondo, Italy; ^3^ Department of Experimental Medicine, Sapienza University of Rome, Rome, Italy; ^4^ Translational Cytogenomics Research Unit, Bambino Gesù Children’s Hospital, IRCCS, Rome, Italy; ^5^ Department of Systems Medicine, University of Rome Tor Vergata, Rome, Italy; ^6^ Division of Metabolic Diseases, Bambino Gesù Children’s Hospital IRCCS, Rome, Italy; ^7^ Diabetology and Growth Disorders Unit, Bambino Gesù Children’s Hospital, IRCCS, Rome, Italy; ^8^ Research Unit of Clinical Immunology and Vaccinology, IRCCS Bambino Gesù Children’s Hospital, Rome, Italy; ^9^ Pediatric Pulmonology and Cystic Fibrosis Unit, Bambino Gesù Children’s Hospital, IRCCS, Rome, Italy; ^10^ Neurosurgery Unit, Bambino Gesù Children’s Hospital, IRCCS, Rome, Italy; ^11^ Department of Pediatrics, Centre Hospitalier Universitaire, CHU, Liège, Belgium; ^12^ Medical Genetics Department, Hospital Pediátrico de Coimbra, Unidade Local de Saúde de Coimbra, Coimbra, Portugal; ^13^ University Clinic of Pediatrics, Faculty of Medicine, University of Coimbra, Coimbra, Portugal; ^14^ Clinical Academic Center of Coimbra, Hospital Pediátrico de Coimbra, Unidade Local de Saúde de Coimbra, Coimbra, Portugal; ^15^ Krishna Institute of Medical Sciences (KIMS Hospital), Hyderabad, India

**Keywords:** NALCN, IHPRF1, CLIFAHDD, channelosome complex, genotype-phenotype correlation, rhythmic behaviors, structural biology

## Abstract

**Introduction:**

Infantile hypotonia with psychomotor retardation and characteristic facies-1 (IHPRF1, MIM#615419) is a rare, birth onset, autosomal recessive disorder caused by homozygous or compound heterozygous truncating variants in *NALCN* gene (MIM#611549) resulting in a loss-of-function effect.

**Methods:**

We enrolled a new IHPRF1 patients’ cohort in the framework of an international multicentric collaboration study. Using specialized *in silico* pathogenicity predictors and *ad hoc* structural analyses, we assessed the mechanistic consequences of the deleterious variants retrieved on NALCN structure and function.

**Results:**

To date 38 different *NALCN* variants have been retrieved from 33 different families, 26 from unrelated and 22 from related patients. We report on five new IHPRF1 patients from four different families, harboring four newly identified and one previously retrieved variant that exhibited a markedly significant functional impact, thereby compromising the functionality of the protein complex.

**Discussion:**

By widening the functional spectrum of biallelic variants affecting the *NALCN* gene, this article broadens the IHPRF1 syndrome’s genotype-phenotype correlation and gives new insight into its pathogenic mechanism, diagnosis, and clinical management.

## 1 Introduction

Infantile hypotonia with psychomotor retardation and characteristic facies-1 (IHPRF1, MIM#615419) is a rare autosomal recessive disorder with an onset at birth or early in infancy, with a typical severe course. Individuals affected by IHPRF1 are generally characterized by moderate-severe hypotonia, global developmental delay, and dysmorphic features (Al-Sayed et al., 2013; Bramswig et al., 2018; Karimi, et al., 2020) IHPRF1 is caused by homozygous truncating variants in *NALCN* gene (MIM#611549) on chromosome 13q33 resulting in a loss-of-function (LoF) effect (Bramswig et al., 2018; Bend et al., 2016) *NALCN* encodes for sodium leak channel, non-selective (NALCN) protein, a voltage-independent, nonselective cation channel that belongs to a family of voltage-gated sodium and calcium channels regulating the resting membrane potential and excitability of neurons with passive, intracellular movement of sodium ions (National Library of Medicine: NCBI, 2024). Novel evidence suggests that NALCN plays a role also in other pathways such as pain sensation (Zhang and Wei, 2023). *NALCN* LoF variants lead to reduced sodium leak causing hyperpolarization of the resting membrane potential explaining the hypoexcitability phenotype that characterizes IHPRF1 disease (Bramswig et al., 2018; Bend et al., 2016). Interestingly, heterozygous pathogenic variants in *NALCN* gene are associated, with another disorder predominantly characterized by congenital contractures of the limbs and face, hypotonia, and developmental delay (CLIFAHDD, MIM#616266) that owns a significant phenotypic overlap degree with IHPRF1, but also shows distal arthrogryposis (Chong et al., 2015).

Other important IHPRF1 features are seizures, brain anomalies (cortical atrophy and/or thin corpus callosum, e.g.,), muscle wasting, strabismus and/or nystagmus, scoliosis, feeding difficulties, constipation, sleep disturbance (secondary to altered circadian rhythm) and breathing abnormalities. Dysmorphic features frequently reported in IHPRF1 are prominent forehead with triangular face, microcephaly, micrognathia, smooth philtrum, thin upper lip, low-set and large ears, slender nose, and strabismus (Al-Sayed et al., 2013; Liu et al., 2013; Bramswig et al., 2018). Periodic breathing and sleep apnea, that usually increase in severity during sleep, are frequently reported both in patients with IHPRF1 and CLIFAHDD (Lozic et al., 2016; Gal et al., 2016; Bourque et al., 2018; Campbell et al., 2018; Maselli et al., 2022; Winczewska-Wiktor et al., 2022) and are generally alleviated by oxygen supplementation or in some cases with support of Non-Invasive Ventilation (NIV) (Lozic et al., 2016).

To date 48 patients have been reported with IHPRF1 (Al-Sayed et al., 2013; Köroğlu et al., 2013; Farwell et al., 2015; Gal et al., 2016; Angius et al., 2018; Bourque et al., 2018; Bramswig et al., 2018; Campbell et al., 2018; Carneiro et al., 2018; Takenouchi et al., 2018; Karimi, et al., 2020; Ope et al., 2020; Maselli et al., 2022; Khan et al., 2022; Abul-Husn et al., 2023; Tehrani Fateh et al., 2023; Susgun et al., 2024) with 38 different *NALCN* variants from 26 unrelated and 22 related patients (33 different families) in homozygosity or compound heterozygosity. Here, we report on five new IHPRF1 patients enrolled in the framework of an international multicentric collaboration study, four with homozygous variants and one with compound heterozygous deleterious variants in *NALCN* gene, in order to: I. further delineate the IHPRF1 genotype-phenotype correlation and its clinical management, II: broaden the spectrum of biallelic variants in *NALCN* gene, and III: assess the mechanistic consequences of these variants on the protein structure and function by resorting to specialized *in silico* pathogenicity predictors and *ad hoc* structural analyses.

## 2 Materials and methods

### 2.1 Study population and data collection

**FIGURE 1:**
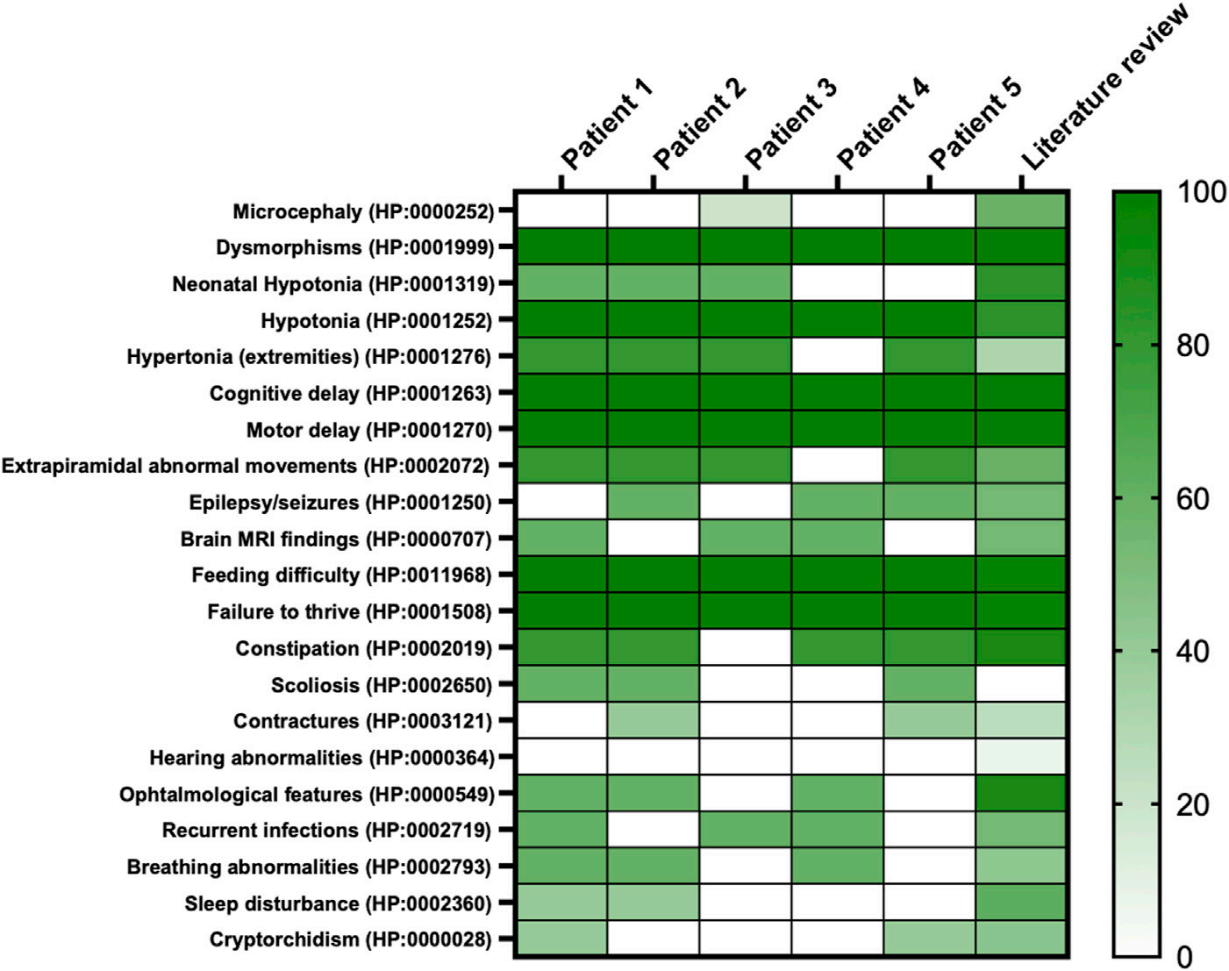
Heatmap of main clinical features frequencies of present cohort compared to literature. On the right, the colored scale depicts patients’ features frequency; from absence and/or very low frequency: light green to very high frequency: deep green. On the left, features retrieved in the whole cohort reported per HPO codes.

This study enrolled five pediatric male patients with *NALCN* deleterious variants. All patients’ data were collected during clinical assessments, after obtaining informed consent and recorded on an anonymized dataset. Patients’ inclusion spanned from May 2023 to March 2024. An extensive literature review on IHPRF1 was conducted by using Embase^®^ and MEDLINE^®^ databases. Data collection included: 1) general information: gender, age, inheritance, nationality; 2) phenotypic description collected by clinical evaluation; 3) laboratory and instrumental investigations: blood tests, EEG, brain MRI, polysomnography. In addition, the main clinical features of the patients with recurrent pathogenic variants were annotated according to the human phenotype ontology (HPO) (Köhler et al., 2021) (Figure 1).

### 2.2 Exome sequencing

Whole Exome Sequencing (WES) was performed on proband and parents’ DNA using the Twist Human Core Exome Kit (Twist Bioscience), according to the manufacturer’s protocol, and sequenced on the Illumina NovaSeq 6000 platform. The BaseSpace pipeline and the Geneyx software LifeMap Sciences were respectively used for the variant calling and annotating variants. Sequencing data were aligned to the hg19 human reference genome.

Variants were reported according to NM_052867.4 and NP_443099.1.

Global minor allele frequency (GMAF) for analyzed variants was reported according to the Genome Aggregation Database (gnomAD) v4.1.0. Based on the guidelines of the American College of Medical Genetics and Genomics (ACMG), a minimum coverage depth of 30X was considered suitable for analysis. Variants were also examined for Qscore and visualized by the Integrative Genome Viewer (IGV) v2.17.4. Clinical interpretation of variants was reported according to the ACMG/AMP 2015 guidelines using the Intervar tool (Li and Wang, 2017).

### 2.3 Biochemical analysis

The biochemical analysis of urinary oligosaccharides both on fresh urine and dried urine spots (DUS), was performed with a UHPLC/MS-MS (Ultra-high performance liquid chromatography tandem mass spectrometry) method. The pre-analytical procedure was obtained without derivatization and using maltoheptaose as internal standard. Urine Samples were ultra-filtered and normalized to a creatinine concentration of 1 mM. The spectrometric analysis was performed in multiple reaction monitoring (MRM) acquisition both in positive and negative mode for all target oligosaccharides (OS) transitions, including the glucose tetrasaccharide Glc4. For each MRM, the median (50th percentile) of several control samples was calculated and results were expressed as MoM. The chromatographic run was 28 min (Heiner-Fokkema et al., 2020).

### 2.4 In-silico assessment of variant pathogenicity

The pathogenic potential of each variant retrieved in our cohort was assessed *in silico* with a pool of variant type-specific software predictors and structural biology methods.

#### 2.4.1 Variant type-specific software predictors

The frameshift variant, p.Tyr1431Leufs*27, causing a premature termination codon (PTC) was evaluated with MutationTasting 2021 (Steinhaus et al., 2021), CADD (Schubach et al., 2024), SIFT-Indel (Hu and Ng, 2013), FATHMM-indel (Ferlaino et al., 2017), and CAPICE (Li et al., 2020). The effect of the nonsense variants, p. (Arg842Ter) and p.Arg855Ter, was evaluated using CADD, DANN (Quang et al., 2015), Eigen (Ionita-Laza et al., 2016), GERP++ (Davydov et al., 2010), LRT (Chun and Fay, 2009), MutationTaster (Schwarz et al., 2010), VEST4 (Carter et al., 2013), MPA (Yauy et al., 2018), and fathmm-MKL (Shihab et al., 2015). In addition, we queried the MetaDome web server (Wiel et al., 2019) to inspect the NALCN tolerance to these variants. The splicing variant, c.2889 + 2T > A, was evaluated using MutationTaster 2021, CADD, Eigen, and MPA, as well as splicing-specific predictors, such as SpliceAI (Jaganathan et al., 2019), Pangolin (Zeng and Li, 2022), MaxEntScan (Yeo and Burge, 2004), and SPiP (Leman et al., 2022). Finally, being the putative pathogenic effect of the missense variant p. (Cys1417Tyr) less straightforward than all the previous variants, it was studied by resorting to both 24 third-party pathogenicity predictors, i.e., CADD, Eigen, MPA, SIFT (Ng and Henikoff, 2003), SIFT4G (Vaser et al., 2016), PolyPhen2 (Adzhubei et al., 2013), FATHMM (Shihab et al., 2013), AlphaMissense (Cheng et al., 2023), REVEL (Ioannidis et al., 2016), ClinPred (Alirezaie et al., 2018), Meta SVM (Kim et al., 2017), Meta LR (Chen et al., 2022), Mistic (Chennen, et al., 2020), DEOGEN2 (Raimondi et al., 2017), DANN, GERP++, LRT, M-CAP (Jagadeesh et al., 2016), MutationTaster (Schwarz et al., 2010), MutationAssessor, MetaLR, PROVEAN (Choi et al., 2012), VEST4, fathmm-MKL, and structural biology methods.

Evolutionary conservation was assessed using GERP++, phyloP, phastCons, and SiPhy tools.

#### 2.4.2 Structural biology

Atomic coordinates of the NALCN protein were retrieved from the AlphaFold Protein Structure Database (Ittisoponpisan et al., 2019), where a high-quality model of the protein was available, with a confidence score (pLDDT) ranging from very high (>90) for the intermembrane ion transport domain to low/very low (<50) for the cytoplasmic α-helical turns and loops. Then, structural damages caused by the missense variant p. (Cys1417Tyr) were investigated using the Missense3D web tool (Schymkowitz et al., 2005). Finally, the stability of the mutant structure was investigated thermodynamically through the BuildModel function implemented in the FoldX algorithm (10.1093/nar/gki387), which was run with default parameters as in Castellana et al. (2021) and Frustaci et al. (2018).

## 3 Results

### 3.1 Clinical cohort description

**FIGURE 2:**
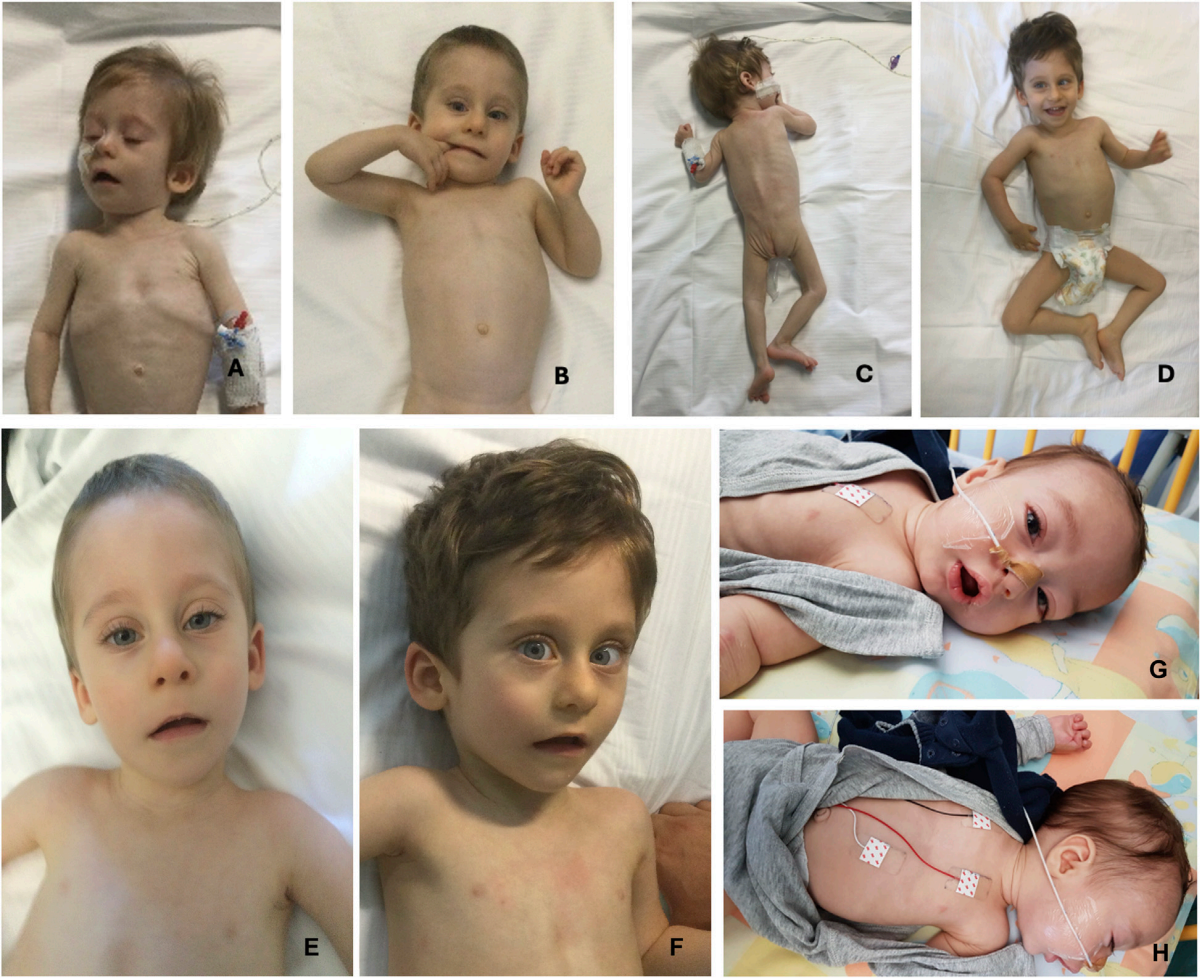
Pictures of two patients from the present cohort. From **(A–D)**, pictures of patient 1 (from 9 months of age to 2 years and 7 months). **(A, B)** pictures depict severe emaciation of the patient and hypotonia whereas before neurosurgery. Pictures **(C, D)** show patient 1’s clinical improvement and weight gain after surgery. **(E, F)** (respectively taken at 18 months and 2 years and 7 months of age) depict the patient 1’s dysmorphic features: triangular face, high forehead, large ears, and mild convergent strabismus. **(G, H)** show patient 4 facial gestalt: triangular face, broad forehead, bitemporal narrowing and the presence of NG tube for enteral feeding.

**FIGURE 3:**
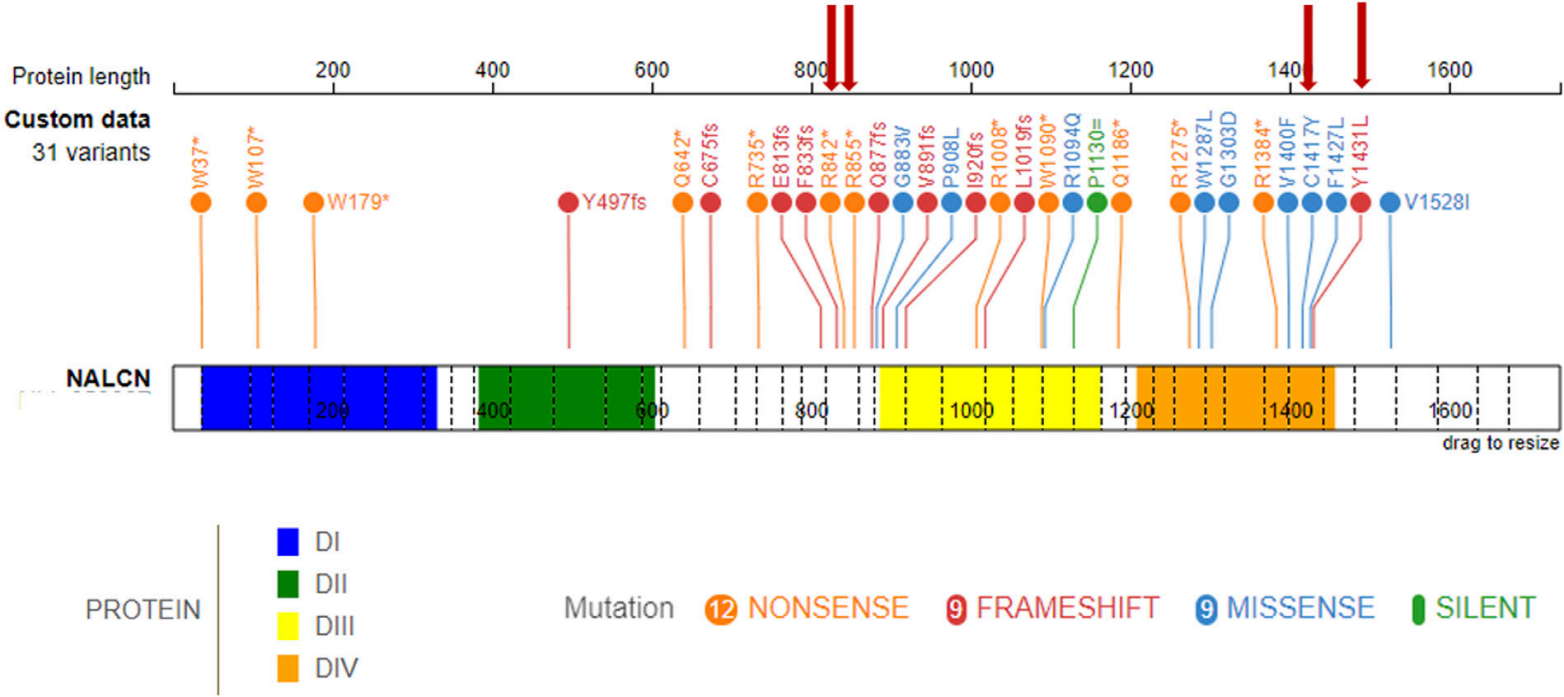
IHPRF1-associated LoF known variants along the NALCN protein. (top) known NALCN variants colored by protein effect (five splicing variants not included). The protein is made up of four homologous domains: DI (blue, 36–331), DII (green, 384–604), DIII (yellow, 887–1163), DIV (orange, 1209–1457), each with six transmembrane segments. Red arrows indicate variants retrieved in the present study.

Main clinical features in the present cohort and their frequencies were compared to the previous literature (Table 1). Available clinical features of patients 1 and 4 are depicted (Figure 2). The main reported features in our cohort were compared with literature (Figure 1), while IHPRF1 patients’ clinical features retrieved are cumulatively reported (Supplementary Table S1). The location of the IHPRF1, both from literature review and those from the present study, are shown along the NALCN protein (Figure 3).

**TABLE 1:** Main clinical features in IHPRF1 patients reported in present cohort and literature review.

Patient ID		Patient 1	Patient 2	Patient 3	Patient 4	Patient 5	Total N = 5 (%)	Literature review
Sex		M	M	M	M	M	5 M	
Age (y = years, m = months)		3 y 5 m	8 y 9 m	1 y	3 y 8 m	13 y	6 y IQR 1–13	
Parental Consanguinity		−	−	+	+	+	3 (60)	59
Normal birth weight		−	+	+	+	−	3 (60)	65
Microcephaly (HP:0000252)		−	−	+	−	−	1 (20)	58
Dysmorphisms (HP:0001999		+	+	+	+	+	5 (100)	
Facial gestalt	TF, BF, BC, LM, LE, LSE, BTN, PC, SN	TF, BF	TF, BF, LSE, TUL	TF, LE	TF, BF, bitemporal flattening of head	TF, TUL	TF 5 (100), BF 3 (60), TUL 2 (40), others 3 (60)	86
Neurologic and developmental features	Neonatal hypotonia (HP:0001319)	+	+	+	−	−	3 (60)	83
	Hypotonia (HP:0001252)	+	+	+	+	+	5 (100)	100
	Hypertonia (extremities) (HP:0001276)	+	+	+	−	+	4 (80)	31
	Cognitive delay (HP:0001263)	+	+	+	+	+	5 (100)	100
	Motor delay (HP:0001270)	+	+	+	++	+	5 (100)	100
	Extrapiramidal abnormal movements (HP:0002072)	+	+	+	−	+	4 (80)	59
	Epilepsy/seizures (HP:0001250)	−	+	−	+	+	3 (60)	54
Brain MRI findings (HP:0000707)		Arachnoid cyst	−	HCC	HCC	−	3 (60)	23
Gastrointestinal features	Feeding difficulty (HP:0011968)	+	+	+	+	+	5 (100)	97
	Failure to thrive (HP:0001508)	+	+	+	+	+	5 (100)	97
	Constipation (HP:0002019)	+	+	−	+	+	4 (80)	92
Muscoloskeletal features	Scoliosis (HP:0002650)	+	+	−	−	+	3 (60)	−
	Contractures (HP:0003121)	−	+	−	−	+	2 (40)	25
Hearing abnormalities		−	−	−	−	−	0 (0)	7
Ophtalmological features		Strabismus, Esotropia	Strabismus Optic atrophy	−	Esotropia	−	3 (60)	90
Other features	Recurrent infections (HP:0002719)	+	−	+	+	−	3 (60)	54
	Breathing abnormalities (HP:0002793)	+	+	−	+	−	3 (60)	42
	Sleep disturbance (HP:0002360)	+	+	−	−	−	2 (40)	62
	Cryptorchidism (HP:0000028)	+	−	−	−	+	2 (40)	44

Main clinical features are reported with HPO codification. “+”: presence of the feature, “−“: absence of the feature. Comparison between our cohort and literature (Suppl. Fig. 2) is reported in the right part of the table. Abbreviations: AT, atrophy; BC, brachycephaly; BF, broad forehead; BTN, Bitemporal narrowing; HCC, hypoplastic Corpus Callosum; LE, large ears; LM, Large mouth; LSE, Low set ears; NA, not available; OA, optic atrophy; PC, pectus carinatum; SN, slender nose; TF, triangular face; TUL, thin upper lip.

**FIGURE 4:**
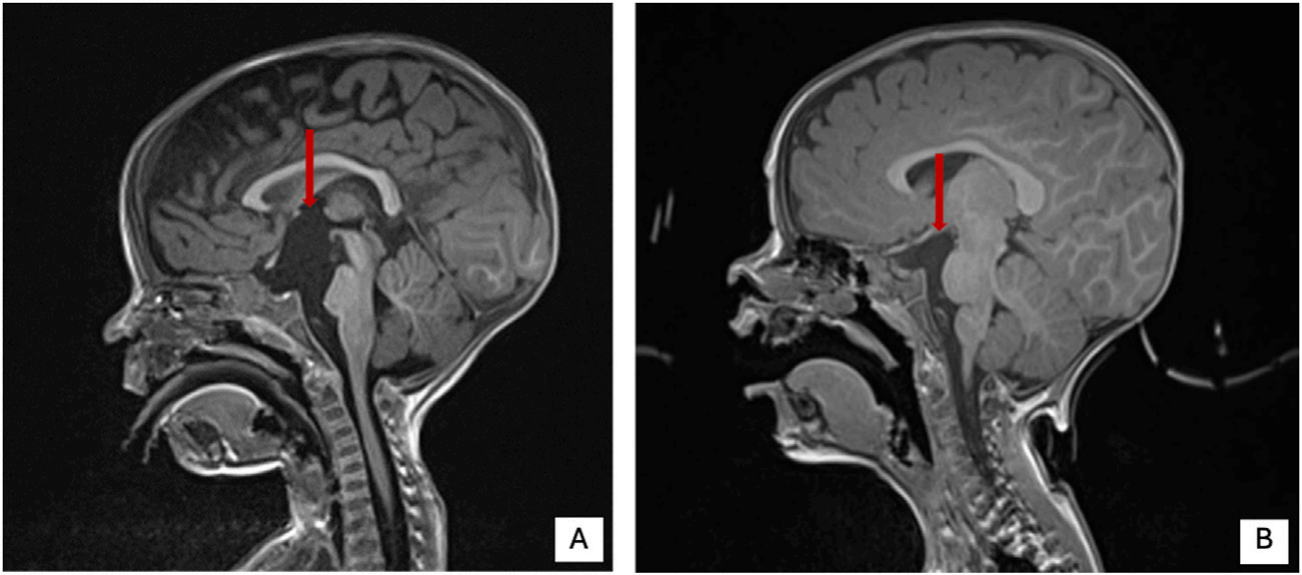
Brain MRI pre and post-surgery in patient 1 of present cohort. **(A)** Brain MRI pre surgery. **(B)** Brain MRI post-surgery. Red arrow shows the cystic lesion.

Patient Report 1: Three-year and five-month-old male, second child of healthy not-consanguineous parents. Family history was not informative for any genetic diseases, his older sister is in good clinical condition. He was born after a regular pregnancy at 38 weeks of gestation from caesarean delivery for previous caesarean section. Anthropometric parameters at birth: weight 2,300 g (-2.5 SD) indicative for a small for gestational age child, length 46 cm (-2.5 SD), and occipitofrontal circumference (OFC) 35 cm (+0.82 SD). APGAR score 9/9. At birth, he showed neonatal hypotonia and dysmorphic somatic features triangular face, high forehead, large ears, and mild convergent strabismus. During follow-up at neurological evaluation were noted: severe psychomotor retardation, predominantly axial hypotonia (not reached the sitting position), hypertonia of the extremities and paroxysmal episodes and rotatory eye movements. The patient came to our observation at the age of 9 months and was admitted to our hospital for the history of failure to thrive (FTT) and finding of a suprasellar cystic lesion detected at brain MRI in another hospital. He presented linear growth during the first 6 months of his life, followed by progressive poor weight gain (Supplementary Figure S1). In consideration of the severe FTT an extensive work-up was performed. To exclude an inborn error of metabolism several examinations were performed resulting normal except for the analysis of urinary oligosaccharides on fresh urine which revealed increased levels of glucose tetrasaccharide (Glc4) in several samples analyzed (Glc4 from 6 to 15 multiple of the medians, MoM, RR < 5). Endocrinology analysis showed reduced levels of IGF1 and IGFBP3 (-2 SD), with normal basal GH. Leptin levels appeared also reduced on multiple measurements (0.57 and 0.06, RR 2.05–5.63 ng/mL). Brain MRI documented the presence of a cyst (diameter 32 mm × 18 mm) which displaced the surrounding structures (pituitary stalk, cerebral peduncles, pons and basilar artery) and modest increase in the size of the lateral ventricles and periencephalic spaces in fronto-temporal regions bilaterally (Figure 4A). Thus, clinical suspicion of Diencephalic Syndrome (DS) was assessed. Endoscopic third ventriculostomy surgery was performed (Figure 4B) with a progressive slight increase in the growth rate and the anatomopathological analysis confirmed the diagnosis of arachnoid cyst, a benign lesion. After surgery, the patient developed iatrogenic hypoadrenalism requiring replacement therapy with hydrocortisone. In the suspicion of sleep apnea, a nocturnal polysomnography examination was performed revealing the presence of periodic breathing, associated with desaturations and normal CO_2_ value. Supplemental oxygen during sleep was prescribed with a progressive amelioration of the periodic breathing (Supplementary Figure S2). Interestingly a slight increase of homovanillic acid and homovanillic/vanilmandelic acid ratio (HVR) values was detected during the investigation for the cyst, showing a progressive normalization after O_2_ supplementation (Supplementary Figure S3). Because of the history of recurrent pneumonia, immunological screening was performed. Partial functional defects of T and B lymphocytes were reported after *in vitro* stimulation with PHA, OKT3, and CpG and a reduction of NK activity was found. He underwent orchidopexy surgery for cryptorchidism at 2 years of age. For the presence of scoliosis, he was prescribed with Cheneau brace. Last auxological parameters at 2-year and 6-month of age: height 85.5 cm (−2 SD), weight 8.5 Kg (−3 SD), OFC 48 cm (−1 SD). Echocardiogram, urinary tract ultrasound, audiological screening and electroencephalography (EEG) resulted normal. WES in trio was then performed revealing the nonsense variant c.2563C > T (p.Arg855Ter) and the splicing variant c.2889 + 2T > A (p.?) in the NALCN gene in compound heterozygosity.

Patient Report 2: Eight-year and 9-month-old male, with a prenatal history of intrauterine growth restriction. At birth, he presented with hypotonia, plagiocephaly, feeding difficulties and poor weight gain. From a neurological standpoint, he has severe psychomotor development delay and epilepsy with an onset at around 6 months. EEG showed initially burst-suppression pattern and later a pre-hypsarrhythmic pattern. He currently has drug-resistant epilepsy, with sporadic seizures. At neurological evaluation was noted severe irritability and involuntary movements with choreoathetosis in the upper limbs and hypertrophy of the thigh muscles, axial hypotonia and hyperreflexia of the limbs. No gait or language acquisition. He is completely dependent on activities of daily living. For the presence of FTT he required nasogastric tube (NG tube) feeding between 6 months and 2 years of age. He also had a dislocated right hip and severe scoliosis (43° Cobb), currently treated with a brace. Surgery is being considered for the future. From a respiratory point of view, at the age of 3 years and 9 months he underwent a polygraph sleep study which showed severe central hypoventilation characterized by constant periodic breathing with central apnea. He underwent non-invasive ventilation at the age of 4 years and 5 months, not tolerate. Parents opted not to continue with non-invasive ventilation. He maintained respiratory stability with no significant infectious complications, but he keeps his habitual irregular breathing pattern and apnea. He also has visual impairment (strabismus and optic nerve hypoplasia) and complaints of constipation. Current auxological parameters: height −2.5 SD, OFC −1.85 SD; weight −3 SD. The analysis of urinary oligosaccharides performed on DUS was normal (Glc4 1.3 MoM, RR < 15). Brain MRI which did not identify any relevant alterations. Finally, WES detected the homozygous nonsense variant c.2524C > T (p.Arg842Ter) in *NALCN* gene.

Patient Report 3: One-year-old male, born from a third-degree consanguineous marriage with an history of delayed achievement of milestones, feeding issues, poor weight gain, and several episodes of respiratory tract infections (in two cases needing hospitalization for Pneumonia). Perinatal and family history were not contributive. On examination, dysmorphisms were noted (triangular face, prominent ears with smooth pinna and hypodontia). Neurological examination showed limb hypotonia. The rest of the systemic examination was not significant. Brain MRI showed hypoplastic corpus callosum. EEG resulted normal. WES performed on the proband and his parents revealed the homozygous missense variant c.4250G > A (p.Cys1417Tyr) in *NALCN* gene. Symptomatic treatment with physiotherapy and occupational therapy was advised to address the concerns in developmental delay.

Patient Report 4: Three-years and eight-month-old male born at 37 weeks of gestational age with a weight of 2,670 g, from a consanguineous family from Morocco with other two children without medical complaints. One episode of hypoglycemia in the neonatal period. Dysmorphic features: bitemporal flattening of the head, wide forehead, triangular face. He presented with a history of feeding difficulties with FTT requiring NG tube and subsequently percutaneous gastric tube (G tube), severe hypotonia, global amyotrophy, severe constipation with frequent fecal impactions. Brain MRI showed a thin corpus callosum. At 8 months, for the presence of spasms he underwent long-term EEG that showed generalized waves-spikes. Valproate and vigabatrin was initiated gradually. Clonazepam was added because of refractory seizures. Patient is now controlled by these three drugs. When he was 2-year-old, he was diagnosed with sleeping apnea syndrome with mild desaturation bur without alveolar hypoventilation. At ophtalmological evaluation finding of esotropia and absence of pursuit movements of the eyes. WES performed on the proband and his parents revealed the homozygous frameshift variant c.4291dupT (p.Tyr1431Leufs*27) variant in *NALCN* gene.

Patient Report 5: Thirteen-year-old male born at term without neonatal complications with a weight of 2,450 g. Dysmorphic features: triangular face, thin upper lip. He presented with a history of severe hypotonia with lower limbs hypertonia and choreic movements. Diagnosis of epileptic encephalopathy initially treated with valproate, phenobarbital and clonazepam, then phenobarbital was switched to lamotrigine because of persistent absence and atonic crisis. Brain MRI was normal. He underwent bilateral inguinal hernia and bilateral cryptorchidism surgical correction at 2 years old. To date he shows good eye contact and interaction with peers, no language acquisition or gait needing a wheelchair. At ophthalmological evaluation finding of strabismus. He presented early with feeding difficulties and gastro-esophageal reflux needing percutaneous gastrostomy. G tube was removed at 2 years of age for parents willing. Currently, he is eating only blended food for difficulties in mastication and swallowing. Over the time he also showed pica eating disorder, scoliosis and severe constipation. WES performed on the proband and his parents revealed the homozygous frameshift variant c.4291dupT (p.Tyr1431Leufs*27) variant in *NALCN* gene.

### 3.2 Biochemical analysis

The urinary oligosaccharides analysis was performed on fresh urine for patient 1 and DUS for patient 2. Two negative MRM transitions of the tetrasaccharide Glc4 and its isomer maltotetraose (M4) were selected as the most characteristic and the Glc4 transition was used for the quantification. For patient 1, we extensively analyzed 7 urine samples at different collection times (2021-2023) obtaining Glc4 increased values of MoM ranging between a minimum of 6 and a maximum of 15 (6, 7.4, 11.8, 13.1, 13.7, 15). Only the last sample analyzed had a normal Glc4 value (4 MoM). The reference value for Glc4 in urine was <5 MoM. For patient 2, we analyzed only 1 DUS sample obtaining the normal value of 1.3 MoM for Glc4. The reference value for Glc4 in DUS was <15 MoM.

### 3.3 *In-silico* assessment of the pathogenicity of variants

**FIGURE 5:**
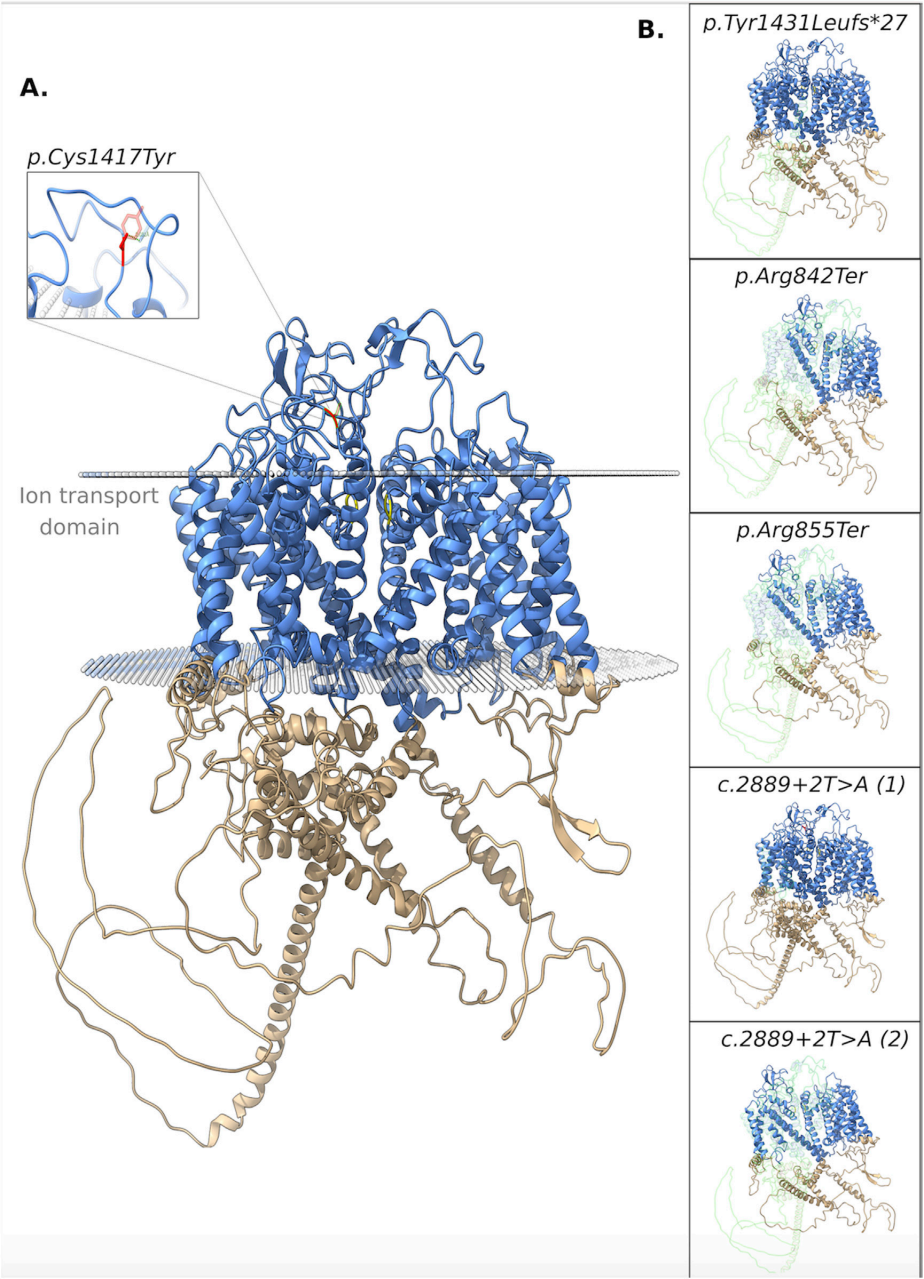
3D structure of the NALCN protein. **(A)** Functional domains: ion transport domain and the selectivity filter highlighted in cyan and yellow, respectively. Focus on the breakage of a disulphide bond. **(B)** Each box shows the impact of a different variant on the protein structure. In particular, the portions of the native protein that are lost due to the frameshift, nonsense, and splicing (the two putative consequences) variants are shown.

The pathogenicity of all variants under examination was evaluated *in silico* using an array of variant type-specific software predictors and structural biology methods. p. (Tyr1431LeufsTer27) caused the formation of a premature termination codon (PTC) 27 residues after the Tyr1431 site. The PTC caused, in turn, the abolishment of a significant portion of the cytoplasmic α-helical turns and loops of the protein and the loss of part of the DIV-S6 transmembrane helix, with a putative disruption of the fundamental activation gate (Figure 5B). While we could not predict whether the protein undergoes degradation upon this mutation, we reported full agreement on its pathogenic effect among five distinct *in silico* indel-specific predictors (Table 2). This variant was not annotated in gnomAD, and was classified as “Likely pathogenic” according to the ACMG/AMP 2015 guidelines. The two nonsense variants p.(Arg842Ter) and p. (Arg855Ter) were considered pathogenic by seven on eight queried predictors and to be phylogenetically highly conserved through 100 vertebrates according to all computed conservation scores (Table 2). Structurally, both variants determine the complete loss of 2 out of 4 transmembrane domains, with dramatic consequences on the protein stability. Variants were reported in gnomAD with different allele frequencies (0,000007435 and 0,0000254, respectively) and also in Clinvar (as “Likely pathogenic” and “Pathogenic,” respectively). Both variants were classified as “Pathogenic” according to the ACMG/AMP 2015 guidelines (Li and Wang, 2017). The splicing variant c.2889 + 2T > A (p.?) is predicted to have a pathogenic effect by all six queried tools that could handle intronic variants (Table 2). Functionally, all splicing-specific queried software packages, i.e., Splice AI, Pangolin, MaxEntScan, and SPiP, predict it to affect the splicing machinery regarding the exon 25. Two splicing events are envisaged. The one less likely (Splice AI score = 0.32) is the loss of the acceptor site of exon 25 located −133 bp from the variant position, thereby causing the exon to skip. The more probable event is the loss of the exon 25 donor site located 2 bp upstream of the variant site (Splice AI score = 1.00), which causes transcription elongation of 24 intronic nucleotides, i.e., GTA​AGT​CTT​TGC​TTA​TTG​CCT​AAA​TGA, before forming a PTC. Structurally, the skipping of exon 25 would affect the voltage-sensor domain 3 (VSD3), with putative consequences on the protein functionality. On the contrary, a so early protein truncation would have consequences on the protein stability like those described above for the nonsense variants in the unlikely case the protein was actually translated (Figure 5B). Variant was not annotated in gnomAD and was classified as “Pathogenic” according to the ACMG/AMP 2015 guidelines (Li and Wang, 2017). Finally, the missense variant p.(Cys1417Tyr) is deemed pathogenic by all queried tools (Table 3). The site is highly conserved through vertebrates and exhibits a MetaDome tolerance score of 0.67, which is compatible with a slightly intolerant site to mutations.

**TABLE 2:** Table summarizing *in silico* pathogenicity predictions for identified nonsense and splicing genetic variants. Each column represents a specific variant, and each row represents a different *in silico* pathogenicity prediction tool.

In-silico predictor	c4291dupT p.(Tyr1431LeufsTer27)	2889+2T>A (?)	c2524C>T p.(Arg842Ter)	c2563C>T p.(Arg855Ter)
Conservation				
GERP++ RS	5.49	—	3.85	4.74
phyloP100way	7.5571	8.947	3.1230	4.6630
phastCons100way	1	1	1	1
SiPhy 29way	—	15.4674	17.3965	15.014
Structural/Functional Impact				
fathmm-MKL	—	D (0.9849)	D (0.8619)	D (0.9602)
LRT	—	—	D (0)	D (0)
Indel-enabled				
MutationTaster21	D (192|2)	D (92|8)	D (194|6)	D (196|4)
CADD-Indel PHRED	D (35)	—	—	—
SIFT-Indel	D (0.858)	—	—	—
FATHMM-indel	D (0.957)	—	—	—
CAPICE	LD (0.9949)	—	—	—
Splicing-enabled				
Splice AI	—	DL (1.00, 2bp); AL (0.32, 133bp)	—	—
Pangolin	—	SL (0.88, 2bp); SG (0.28, 31bp)	—	—
MaxEntScan	—	8.00 (−102.25%)	—	—
SPiP	—	98.41%	—	—
Overall Pathogenic Potential				
MutationTaster	—	A (1)	A (1)	A (1)
CADD PHRED	—	D (34)	D (36)	D (47)
DANN	—	D (0.9948)	D (0.9967)	D (0.9981)
Eigen-PC-raw	—	0.996	N (−0.1061)	N (0.7699)
VEST4	—	—	D (0.958)	D (0.931)

The information within each table cell is formatted as “Pred (score),” where possible. Pred can be: D, deleterious; A, disease causing automatic; LD, likely deleterious; N, neutral. Splice AI: DL/AL (score, bp), donor loss/acceptor loss (score [0-1], distance from the variant); Pangolin: SL/SG (score, bp), splice loss/gain (score [0-1], distance from the variant). MaxEntScan: score (%variation between wild-type and mutant). SPiP: score [0-1]. For all splicing predictors, the higher the score more likely the splicing impairment.

**TABLE 3:** Table summarizing *in silico* pathogenicity predictions for the identified missense variant. Each row represents a different *in silico* pathogenicity prediction tool.

In-silico predictor	c4250G>A p. (Cys1417Tyr)
Conservation-based	
GERP++ RS	5.51
phyloP100way	7.4720
phastCons100way	1
SiPhy 29way	19.7859
Structural/Functional Impact	
Polyphen2 HDIV	D (1)
SIFT	D (0.001)
SIFT4G	D (0)
MutationAssessor	H (3.825)
PROVEAN	D (−8.83)
FATHMM	D (−4.94)
fathmm-MKL	D (0.9856)
LRT	D (0)
Overall Pathogenic Potential	
MutationTaster	D (1)
CADD PHRED	D (26.8)
DANN	D (0.9976)
DEOGEN2	D (0.9745)
Eigen-PC-raw	D (0.9311)
M-CAP	D (0.6640)
MetaLR	D (0.9749)
VEST4	D (0.962)
AlphaMissense	D (0.9988)

The information within each table cell is formatted as “Pred (score),” where possible. Pred can be: D, deleterious; H, high impact.

From a structural standpoint and considering that the NALCN is a four-domain (domains DI to DIV, Figure 3) ion channel characterized by a transmembrane region (in blue in Figure 5A), where each domain includes six transmembrane segments, and two soluble regions that protrude in both extracellular and cytoplasmic sides (Kschonsak et al., 2020), the missense p. (Cys1417Tyr) variant is located into one of these extracellular soluble loops (Figure 5A), where an elaborate network of disulfide-bond from each domain creates an appendage above the pore selectivity filter. As reported by Missense3D, this residue substitution can be classified as *damaging* due to the disruption of one of these fundamental disulphide bonds, in particular between the Cys1417 and the Cys1405. We assessed the protein stability using the FoldX algorithm to further confirm its thermodynamic impact. Following free energy calculation, we estimated a clear ΔΔG increase (ΔΔG_p.(Cys1417Tyr)_ = 10.9587 + 0.005 kcal/mol), compatible with a highly destabilizing effect. This variant, which was reported in gnomAD with a global AF of 0,000003424, was classified as a Variant of Uncertain Significance (VUS) according to the ACMG/AMP 2015 guidelines (Li and Wang, 2017).

## 4 Discussion

Pathogenetic variants of the *NALCN* gene, in homozygosity or compound heterozygosity with a putative LoF role, are associated with IHPRF1, that is inherited in an autosomal recessive manner, in contrast to heterozygous pathogenic variants in NALCN with a GoF effects, that are associated with CLIFAHDD. According to Monteil et al. (2024), variants reported in CLIFAHDD are mostly missense and are concentrated within the pore-forming S5 and S6 helices, while IHPRF1 variants are broadly distributed and the majority are truncating variants (nonsense, frameshift or deletions) (Monteil et al., 2024). LoF variants in IHPRF1 seem to cause hyperpolarization of the resting membrane potential explaining the hypoexcitability phenotype that characterizes IHPRF1 disease with profound hypotonia (Bend et al., 2016; Bramswig et al., 2018). On the other hand, most of the literature seems to indicate that the putative mechanism underlying CLIFAHDD is the gain of function of NALCN explaining the presence of arthrogryposis caused by hyperexcitability of the motor unit, secondary to the depolarization of the resting membrane potential, resulting in a hypercontracted phenotype (Bramswig et al., 2018; Bouasse et al., 2019). However, according to Bend et al. (2016), some variants reported in CLIFAHDD, seem to have a loss of function mechanism acting as dominant negative in functional assays on *C. elegans* (CLIFAHDD-dominant Antimorphic) (Bramswig et al., 2018).

In this study, we reported five male patients from four different families of whom four carried homozygous variants (patient 2, 3, 4, and 5) and one (patient 1) inherited a compound heterozygosity in the *NALCN* gene. Only the nonsense variant c.2563C > T (p.Arg855Ter) retrieved in patient 1 was already previously reported by Karimi et al. (2020), the others (1 splicing, 1 missense, 1 frameshift, and 1 nonsense) are retrieved for the first time. To date, 36 different variants have been reported in the literature in homozygosis or compound heterozygosis associated to IHPRF1, and among them, 12 nonsense, 9 missense, 9 frameshift, 5 splicing, and 1 synonymous variant (Figure 3; Supplementary Table S2). Frequencies of the main clinical features are comparable with those previously reported in literature (Table 1; Figure 1). Considering the clinical phenotype of our patients is it possible to observe that the whole cohort experienced feeding difficulties (needing in some cases NG or G tube) and FTT. This finding is consistent with the literature on IHPRF1 (Al-Sayed et al., 2013; Bramswig et al., 2018; Campbell et al., 2018). Interestingly, patient 1, after 6 months of linear growth, presented with severe FTT, in the presence of adequate caloric intake. Finding at brain MRI of a large arachnoid cyst in the suprasellar region needing surgical corrections led to the clinical diagnosis of DS. DS is a rare cause of FTT in infants associated with central nervous system tumors in the hypothalamic region. Clinical presentation includes severe emaciation, lipodystrophy, normal linear growth, and histories of poor weight gain starting at about 6 months of age despite having normal caloric intake (Burr et al., 1976; Trapani et al., 2022). Other features described but more inconstant are hyperalertness, hyperkinesia, hydrocephalus, nystagmus, visual field defects, and vomiting. The DS is characterized by the absence of specific laboratory findings related to hypothalamic dysfunction and it is often a diagnosis of exclusion after a long differential diagnostic work-up (Burr et al., 1976; Fleischman et al., 2005). MRI is considered the gold standard for the diagnosis of a cerebral mass causing DS (Hoffmann et al., 2014; Trapani et al., 2022). Treatment includes surgical resection, radiation therapy, and chemotherapy, and it is often followed by weight gain (Kim, 2012; Tosur et al., 2017). Significantly, during the diagnostic workup for patient 1 an elevation of urinary oligosaccharides with increased levels of glucose tetrasaccharide (Glc4) was reported. Glc4 (Glca1-6Glca1-4Glca1-4Glc), is a glycogen-derived dextrin that correlates with the extent of glycogen accumulation in skeletal muscle (Young et al., 2009). Glc4 is described in the literature as the most sensitive biochemical biomarker of Glycogen storage diseases (GSDs) type II (acid α-glucosidase deficiency, Pompe disease, PD, OMIM #232300) a metabolic disorder affecting mainly the liver and muscle. It was described in patients performing a whole-body muscle magnetic resonance imaging a correlation between muscle strength, the amount of fatty infiltration in muscle, and urinary Glc4 (Khan et al., 2020). The degree of elevation appears to correlate with the severity of the clinical phenotype and the stage of Pompe disease (Piraud et al., 2020; Heiner-Fokkema et al., 2020), but it can be elevated in other conditions associated with increased glycogen storage, including certain leukemias and sarcomas, Duchenne muscular dystrophy and acute pancreatitis (Young et al., 2009). Glc4 levels were markedly increased also in three disorders belonging to the autophagy machinery: Vici syndrome, Yunis-Varon syndrome, and Danon disease, all sharing a cardiomuscular involvement with increased glycogen storage and variable vacuoles accumulation detectable on light microscopy at the level of skeletal and cardiac muscle (Semeraro et al., 2021; Mastrogiorgio et al., 2021). Since muscular wasting and severe hypotonia are frequently reported in IHPRF1, we hypothesized that Glc4 could be a marker of the neuromuscular damage in this condition, so we measured Glc4 also in patient 2 but the examination tested negative. Interestingly, the urinary glucosaccharides exam was repeated in patient 1 after neurosurgery and amelioration of the growth pattern, with Glc4 levels normalization. This finding led us to hypothesize that Glc4 elevations may be considered as a DS biomarker which could be associated likewise retrieved in patient 1, with severe muscular wasting and FTT. Of note leptin levels in our patient were suppressed in several determinations, with a slight increase after surgery and weight gain. Leptin is a pleiotropic hormone primarily secreted in adipose tissue by adipocytes and is implicated in a wide range of biological functions that control different processes such as the regulation of body weight and energy expenditure, stimulates thermogenesis, and reduces appetite, reproductive function, immune response, and bone metabolism (Greco et al., 2021). In recent years, leptin has been widely studied in obesity and anorexia nervosa, and its plasma levels are related to BMI (Miller, 2011). In literature, DS is associated in large case series with low levels of leptin and concomitant increase of ghrelin (Brauner et al., 2006; Trapani et al., 2022). In some cases, reductions in leptin levels have been reported after surgical lesions’ excision probably due to a direct secretion by the tumor mass (Velasco et al., 2014). In humans, decreasing leptin concentrations in response to food deprivation are responsible for the starvation-induced suppression of the hypothalamic-pituitary-gonadal axes (Sainsbury et al., 2002). Low levels of leptin have been observed also in patients with FTT (Shaoul et al., 2003). According to Monteil et al. (2024) leptin seems to act as a positive regulator of NALCN. Recent evidence demonstrated how leptin is a potent respiratory stimulant, by activating NALCN to depolarize neurons type 1 of the solitary tract (NTS) that express the long form of leptin receptor (LepRn), providing a glutamatergic pathway that relays excitation to premotor breathing control areas in the ventral respiratory group (rVRG) which harbors bulbospinal inspiratory premotor neurons (Do et al., 2020). Do et al. (2020) also showed that the leptin-induced depolarization in type-1 cells was abolished in NALCN-cKO mice (selective deletion of NALCN in LepRb neurons), with an increase of breathing irregularities, concluding that NTS LepRb neurons contribute to the stability of the respiratory pattern and reduce the occurrence of both central and obstructive apneas. Moreover, previous studies demonstrated that injection of leptin into NTS of rats stimulates respiratory output (Inyushkina et al., 2010). This observation led us to speculate on the hypothesis that low levels of leptin in the context of IHPRF1 or CLIFAHDD can worsen the clinical picture of these patients. In this perspective, good management of FTT and weight loss could ameliorate the phenotype of the patients with IHPRF1, in particular for periodic breathing. Patient 1 presented a progressive increase of leptin values after surgery and weight gain (from 0.06 ng/mL to 1.18 ng/mL, even if still under reference value) and this was accompanied by improvement of clinical condition and periodic breathing. The patient had already started oxygen supplementation so it is difficult to assess if the increase in leptin levels may have improved this aspect. We suggest that further studies on leptin levels in a larger cohort of patients with IHPRF1 and CLIFAHDD with FTT are needed to better assess the role of this hormone in the variability of clinical features.

Breathing abnormalities are frequently reported in both patients with IHPRF1 (Campbell et al., 2018; Maselli et al., 2022) and CLIFAHDD (Gal et al., 2016; Lozic et al., 2016; Bourque et al., 2018; Winczewska-Wiktor et al., 2022). NALCN mutant mice have a severely disrupted respiratory rhythm interrupted by periods of apnea and die within 24 h of birth. Breathing pattern in NALCN null mice is characterized by apnea for 5 s followed by a burst of breathing for 5 s at a rate of 5 episodes of apnea per minute imputable to a complete loss of electrical activity from the fourth cervical root (C4) that innervates the diaphragm with rhythmic electrical signals (Lu et al., 2007). Respiratory rhythms are generated by inspiratory neurons in the pre-Bötzinger complex (preBötC) network of the medulla generating the rhythmic output to cranial and spinal motor neurons. This network is modulated by Substance P that exerts excitatory effects in preBötC neurons by modulating a nonselective cation channel with the properties of NALCN (Lozic et al., 2016; Monteil et al., 2024). Moreover, NALCN is expressed in CO_2_/H (+)-sensitive neurons of retrotrapezoid nucleus (RTN) that regulate breathing. *In vivo*, RTN-specific knockdown of *NALCN* reduced CO_2_-evoked neuronal activation and breathing (Shi et al., 2016). The main treatment for periodic breathing and apnea in the reported cases is based on oxygen supplementation at night (Lozic et al., 2016; Campbell et al., 2018), in some cases requiring NIV (Maselli et al., 2022). Tracheostomy is rarely required (Lozic et al., 2016; Vivero et al., 2017). In our cohort, we reported three patients with breathing abnormalities, patient 1 with periodic breathing and severe central apneas requiring oxygen supplementation, patient 2 with periodic breathing and severe central apneas requiring NIV (discontinued on parent’s decision) and patient 4 with mild central apneas not requiring medical treatment. Interestingly, an increase in urinary homovanillic acid, vanilmandelic acid and NSE values was observed over time in patient 1 during the workup for the presence of brain cysts (before detecting a benign lesion at anatomopathological evaluation) that did not normalize after surgery. Conversely, after the introduction of oxygen supplementation, a decrease in the respective values was observed, with normalization of the homovanillic/vanilmandelic acid ratio (Supplementary Figure S3). Thus, it is possible to hypothesize that the progressive normalization of the urinary homovanillic acid, vanilmandelic acid and their ratio, could be associated to the amelioration of periodic breath, after the low flow oxygen therapy. In literature, several studies suggested an alteration of the sympathetic nervous system with an increase in urinary norepinephrine levels in apneic patients (Dimsdale et al., 1995). To date, most of the studies evaluated this relation in obstructive sleep apnea (OSA). In a recent pediatric systematic review, it was reported that catecholamine levels can be pointed out as a marker for sympathetic nervous system excitability in OSA, and correlate with specific clinical, cardiovascular, neurobehavioral, and metabolic parameters, contributing to increased morbidity (Sica et al., 2021). It is shown that continuous positive airway pressure (CPAP) treatment in patients with OSA reduces catecholamine levels and blood pressure (Green et al., 2021). Sympathetic nervous system activation was observed also in patients with central sleep apnea (McLaren et al., 2019). Recurrent infections, in particular respiratory tract infections, are another important clinical finding among IHPRF1 and CLIFAHDD patients (Bramswig et al., 2018; Karimi, et al., 2020; Tehrani Fateh et al., 2023). In our cohort, we detected two patients with recurrent infections, patient 1 with frequent respiratory infections requiring antibiotic prophylaxis, and patient 3 with multiple episodes of pneumonia. According to Bramswig et al. (2018), recurrent infections in the cohort were not associated with immunological alterations (specific information on test performed was not reported) and were correlated with FTT. Recently, Tehrani Fateh et al. (2023) described a IHPRF1 patient with recurrent urinary tract infections hypothesizing that abnormal contraction of detrusor muscle due to NALCN LoF could lead to incomplete emptying of the bladder with an increased risk of infections. However, patient 1 of the present cohort was found with partial functional defects of T and B lymphocytes that were reported after *in vitro* stimulation with PHA, OKT3, and CpG and a reduction of NK activity. According to Karimi et al. (2020), male individuals seem to display a more severe phenotype when compared to female individuals, even in the same family. Interestingly, estrogen and progesterone play a role in the regulation of *NALCN* expression in human myometrial muscle cells suggesting a sex-driven regulation for NALCN. All patients in the present cohort are males and some features such as epilepsy and breathing abnormalities are slightly higher compared to the literature review (which includes both male and female individuals), confirming that the male sex worsens the phenotype in this disease.

Finally, all variants cause a markedly significant functional impact, thereby compromising the functionality of the protein complex, as indicated by *in silico* and structural analyses. Variants introducing premature stop codon may act at different levels, mutant transcripts might undergo nonsense-mediated mRNA decay, or, alternatively, the proteins could be produced but may be severely affected in terms of function and interaction with other elements of the complex. When considering patients harboring nonsense truncating variants in codons 842 (p.Arg842Ter) and 855 (p.Arg855Ter) in the present cohort, namely, patient 1 and patient 2 and the two siblings described by Karimi et al. (2020), it is interesting to observe that all of these four patients seems to display breathing abnormalities that in our two cases are severe, needing in one case oxygen supplementation (patient 1) and NIV (patient 2). We also reported a new missense variant in homozygosity, p.Cys1417Tyr, located into an extracellular soluble loop of the protein. To date, missense variants are less frequently reported as pathogenic, and, only one missense variant c.3860G > T, (p.Trp1287Leu) was reported in homozygosity in three patients from the same family localizing on a site close to the outer region of the channel, with likely milder functional consequences (Al-Sayed et al., 2013). Close to the missense homozygous variant p.Cys1417Tyr described in the present work, a missense variant c.4281C > A, p.Phe1427Leu in compound heterozygosity with a splicing variant (c.4281C > A and c.4103 + 2T > C) in two siblings located in the predicted S6 pore forming segment of domain IV was reported (Bramswig et al., 2018). Of interest, the phenotype of these patients, along with the clinical picture of patient 3 from the present cohort harboring the missense homozygous variant c.4250G>A p.Cys1417Tyr seems to be milder compared with patients harboring truncating variants in *NALCN*. Further studies and functional *in vitro* analyses will be necessary to characterize the molecular effects that truncating and missense variants have at the molecular level to enable more accurate genotype-phenotype correlations.

## 5 Conclusion

In this study, we focused on WES, *in silico* pathogenicity predictors, and structural analyses to examine genetic variants retrieved from a new IHPRF1 patients’ cohort. We described five new IHPRF1 patients from four different families, harboring four newly identified and one previously retrieved variant whose deep investigation exhibited a markedly significant functional impact, thereby compromising the functionality of the NALCN complex. The present findings, taken together, widen the pathogenic mechanism and functional spectrum of biallelic variants affecting the *NALCN* gene, as well as the IHPRF1 syndrome diagnosis and genotype-phenotype correlation.

## Data Availability

The original contributions presented in the study are included in the article/Supplementary Material, further inquiries can be directed to the corresponding author.
