# Supplementary material for: Widening the infantile hypotonia with psychomotor retardation and characteristic Facies-1 Syndrome’s clinical and molecular spectrum through NALCN in-silico structural analysis
Source: Front Genet. 2024 Dec 11;15:1477940. doi: 10.3389/fgene.2024.1477940 (PMC11668739; doi:10.3389/fgene.2024.1477940)
Supplement: Supplementary file 1 [file Table1.docx]

**Supplemental Table 1:** Main clinical features of patients with IHPRF1 reported in literature. Papers of Farwell et al. (2015) and Abul-Husn et al. (2023) were not considered because they lack specific information on IHPRF1 patients. NA information was not considered for total frequencies. “+”: presence of the feature, “-“: absence of the feature. Abbreviations: AT: atrophy, BC: brachycephaly, BF: broad forehead, BTN: Bitemporal narrowing, HCC: hypoplastic Corpus Callosum, LE: large ears, LM: Large mouth, LSE: Low set ears, NA: not available, OA: optic atrophy, PC: pectus carinatum, SN: slender nose, TF: triangular face, TUL: thin upper lip.

| ***Main clinical features in IHPRF1 patients reported in literature*** | | | | | | | | | | | | | | | | | |
| --- | --- | --- | --- | --- | --- | --- | --- | --- | --- | --- | --- | --- | --- | --- | --- | --- | --- |
| References |  | ***Al-Sayed***  ***(2013)*** | ***Koroglu (2013)*** | ***Gal (2016)*** | ***Takenouchi (2018)*** | ***Campbell (2018)*** | ***Bramswing (2018)*** | ***Angius (2018)*** | ***Bourque (2018)*** | ***Carneiro (2018)*** | ***Karimi (2020)*** | ***Ope (2020)*** | ***Khan (2022)*** | ***Tehrani Fateh (2023)*** | ***Susgun (2024)*** | ***Total literature cohort***  ***N (%)*** |  |
| Number of patients |  | 6 | 2 | 3 | 2 | 3 | 16 | 2 | 1 | 1 | 1 | 2 | 1 | 3 | 2 | 45 |  |
| Sex |  | F (3/6) | F | F (2/3) | F | F (2/3) | F (8/16) | F (1/2) | M | F | F | F (1/2) | M | F (2/3) | F | 24 F 21 M |  |
| Parental Consanguinity |  | + | + | + | - | - | + (8/16) | - | + | NA | + | - | + | + | NA | 25/42 (59) |  |
| Normal birth weight |  | + | + | + | - | - | + (10/16) |  | - | NA | NA | + | + | + | NA | 27/41 (65) |  |
| Microcephaly |  | NA | + | NA | - | - | + (12/16) | NA | + | + | + | + (1/2) | NA |  | NA | 18/31 (58) |  |
| Dysmorphisms |  | + | + | + | + | + (2/3) | + | + | - | + | + | + | + | + (1/3) | NA | 39/45 (86) |  |
| Facial gestalt | TF, BF, BC, LM, LE, LSE, BTN, PC, SN | BF, TF, BC,  SN | BF, LSE, PC | Myopathic face | Tented mouth | Myopathic face, bitemporal narrowing | TF, BF, fine hair, large ears | TUL, BTN, high nasal bridge, LSE, LM | NA | Palpebral ptosis | TF, micrognathia, smooth philtrum, LSE, LM, PC | PC, dolichocephaly | NA | TF, LSE | Na | - |  |
| Neurologic and developmental features | Neonatal hypotonia | + | - | + (1/3) | + | + | + | + | + | NA | NA | + | NA | + (2/3) | + | 35/42 (83) |  |
|  | Hypotonia | + | + | + | + | + | + | + | + | + | + | + | NA | + | + | 44/44 (100) |  |
|  | Hypertonia (extremities) | + | NA | + | NA | - | + (2/16) | - | - | - | + | - | NA | - | - | 12/38 (31) |  |
|  | Cognitive delay | + | + | + | + | + | + | + | + | + | + | + | + | + | + | 45/45 (100) |  |
|  | Motor delay | + | + | + | + | + | + | + | + | + | + | + | + | + | + | 45/45 (100) |  |
|  | Extrapiramidal abnormal movements | + | + | + (2/3) | NA | - | + (12/16) | + | - | NA | - | - | + | - | - | 25/42 (59) |  |
|  | Epilepsy/seizures | + (3/6) | + | + (2/3) | - | - | + (7/16) | + | + | NA | - | + (1/2) | + | + | + | 24/44 (54) |  |
| Brain MRI | AT, Other | Abnormal (1/6) | AT | Abnormal  (1/3) | Normal | Normal | Abnormal (4/16) | Abnormal | Normal | NA | Abnormal | NA | NA | Normal (1/3) | NA | Abnormal 9/39 (23) |  |
| Gastrointestinal features | Feeding difficulty | + | + | + | + | + | + (15/16) | + | + | + | + | + (1/2) | + | + | NA | 41/42 (97) |  |
|  | Failure to thrive | + | + | + | + | + | + | + | + | + | NA | + (1/2) | + | + | NA | 41/42 (97) |  |
|  | Constipation | + | + | + | + | + | + (14/16) | + | + | NA | + | - | NA | + | NA | 38/41 (92) |  |
| Muscoloskeletal features | Contractures | + | NA | NA | - | - | + (2/16) | - | - | NA | + | - | NA | NA | NA | 9/35 (25) |  |
| Hearing abnormalities |  | - | Progressive deterioration | NA | NA | - | NA | NA | NA | - | NA | NA | NA | - | NA | 1/13 (7) |  |
| Ophtalmological abnormalities | SB, ES, NY, OA | NY, SB | NY, OA | NY | ES | SB, hypermetropia | Strabismus (12/16) | Strabismus | Strabismus, NY | Optic atrophy, myopia, decreased visual acuity | Strabismus | Strabismus, esotropia | NA | Strabismus (2/3), visual impairment (1/3) | NA | 37/41 (90) |  |
| Other features | Recurrent infections | NA | NA | NA | NA | NA | + (9/16) | NA | NA | NA | + | - | NA | + (2/3) | NA | 12/22 (54) |  |
|  | Breathing abnormalities | NA | - | + (2/3) | NA | NA | + (5/16) | NA | + | NA | + | + | NA | + (1/3) | NA | 12/28 (42) |  |
|  | Sleep disturbance | NA | NA | NA | + (1/2) | NA | + (10/16) | + | + | NA | + | - | NA | NA | NA | 15/24 (62) |  |
|  | Cryptorchidism | NA | + (1/2) | NA | NA | NA | NA | NA | + |  | - | + | NA | - |  | 4/9 (44) |  |

| ***Spectrum of variants in patients with IHPRF1 reported in literature and present cohort*** | | | | | | | | |
| --- | --- | --- | --- | --- | --- | --- | --- | --- |
| **Patient/Reference** | **cDNA** | **Protein** | **Protein change** | **Exon** | **Location** | **Type of mutation** | **Class** | **Patients and Gender** |
| **Compound Heterozygosis variants** |  |  |  |  |  |  |  |  |
| Campbell et al, 2018 | c.110G>A (Mother)  c.2723C>T (Father) | p.Trp37Ter  p.Pro908Leu | W37* P908L | 3 24 | N-Term  DIII | N  M | III | 1 M 1 F: Sibs |
| Maselli et al, 2022 | c.1267-924_1434+2024del (Mother)  c.3022C>T (Father) | ? p.Arg1008Ter | ?  R1008* | 13 26 | DIII | L  N | IV V | 1 M |
| Farwell et al, 2016 | c.2203C>T (Parent 1) c.4197+1G>A (Parent 2) | p.Arg735Ter ? | R735* ? | 19 38 | DII-DIII loop Domain IV | N  L | V | 1 NA |
| **Present report Pt 1** | **c.2889+2T>A (Mother)  c.2563C>T (Father)** | **?  p.Arg855Ter** | **?  R855*** | **26 22** | **DIII  DII-DIII loop** | **L  N** | **IV**  **V** | **1 M** |
| Bramswig et al, 2018 | c.3022C>T (Mother)  c.2629del (Father) | p.Arg1008Ter p.Gln877fs | R1008* Q877fs | 26 23 | DIII  DII-DIII loop | N F | **V** | 1 F |
| Campbell et al, 2018 | c.3281G>A (Mother)  c.2563C>T (Father) | p.Arg1094Gln  p.Arg855Ter | R1094Q R855* | 29 22 | DIII  DIII | M N | V | 1 F |
| Angius et al, 2018 | c.3823C>T (Mother)  c.2495_2496insTCATA (Father) | p.Arg1275Ter  p.Phe833HisfsTer40 | R1275* F833fs | 34 22 | DIV  DII-DIII | N F | V | 1 M 1 F: Sibs |
| Bramswig et al, 2018 | c.4281C>A (Mother)  c.4103+2T>C (Father) | p.Phe1427Leu  ? | F1427L ? | 38 37 | DIV  DIV | M L | V | 2 M: Sibs |
| Carneiro et al, 2018 | c.4582G>A (Parent 1) c.4198G>T (Parent 2) | p.Val1528Ile  p.Val1400Phe | V1528I V1400F | 40 38 | C term  DIV | M M | III | 1 F |
| **Homozygosis variants** |  |  |  |  |  |  |  |  |
| Bramswig et al, 2018 | c.321G>A | p.Trp107Ter | W107* | 4 | DI | N | V | 1 M 1 F: Sibs |
| Bramswig et al, 2018 | c.537del | p.Trp179Ter | W179* | 6 | DI | N | V | 1 F |
| Takenouchi et al, 2018 | c.1267-2A>G | p.? | ? | 12 | DI | L | V | 1 F |
| Tehrani Fateh et al, 2023 | c.1434 + 1G > A | p.? | ? | 12 | DI | L | V | 1 F |
| Al-Sayed et al, 2013 | c.1489del | p.Tyr497fs | Y497fs | 13 | DI | F | V | 2 M: Sibs ; 1 M |
| Koroglu et al, 2013 | c.1924C>T | p.Gln642Ter | Q642* | 16 | DII-DIII loop | N | V | 1 F 1 M: Sibs |
| Takenouchi et al, 2018 | c.2022_2023delAT | p.Cys675fs | C675fs | 17 | DII-DIII loop | F | V | 1 F |
| Ope et al, 2020 | c.2203C>T | p.Arg735Ter | R735* | 19 | DII-DIII loop | N | V | 1 M 1 F: sibs |
| Bramswig et al, 2018 | c.2435dup | p.Glu813fs | E813fs | 21 | DII-DIII loop | F | V | 1 F |
| **Present report Pt 2** | **c.2524C>T** | **p.Arg842Ter** | **R842*** | **22** | **DII-DIII loop** | **N** | **IV** | **1 M** |
| Karimi et al, 2020 | c.2563C>T | p.Arg855Ter | R855* | 22 | DII-DIII loop | N | V | 1 F |
| Tehrani Fateh et al, 2023 | c.2648G>T | p.Gly883Val | G883V | 24 | DIII | M | III | 1 F |
| Bramswig et al, 2018 | c.2671del | p.Val891fs | V891fs | 24 | DIII | F | IV | 1 M 1 F: sibs ; 1 M |
| Bramswig et al, 2018 | c.2758del | p.Ile920fs | I920fs | 25 | DIII | F | V | 1 F |
| Bramswig et al, 2018 | c.2889+3_2889+6del | ? | ? | 26 | DIII | L | V | 1 F |
| Abul-Husn et al, 2023 | c.3022C>T | p.Arg1008Ter | R1008* | 26 | DIII | N | V | 1 NA |
| Bramswig et al, 2018; Susgun et al, 2024 | c.3056dupT | p.Leu1019fs | L1019fs | 27 | DIII | F | V | 1 M 1 F: Sibs ; 2 F: Sibs |
| Tehrani Fateh et al, 2023 | c.3269G>A | p.Trp1090Ter | W1090* | 28 | DIII | N | V | 1 M |
| Gal et al, 2016 | c.3390G>A | p.Pro1130= | P1130= | 30 | DIII | S | V | 2 F 1 M: Sibs |
| Bramswig et al, 2018 | c.3556C>T | p.Gln1186Ter | Q1186* | 31 | DIII-DIV loop | N | V | 1 F |
| Bourque et al, 2018 | c.3823C>T | p.Arg1275Ter | R1275* | 34 | DIV | N | V | 1 M |
| Al-Sayed et al, 2013 | c.3860G>T | p.Trp1287Leu | W1287L | 34 | DIV | M | V | 3 F: Sibs |
| Khan et al, 2022 | c.3908C>T | p.Gly1303Asp | G1303D | 35 | DIV | M | V | 1 M |
| Bramswig et al, 2018 | c.4150C>T | p.Arg1384Ter | R1384* | 37 | DIV | N | V | 1 M |
| **Present report Pt 3** | **c.4250G>A** | **p.Cys1417Tyr** | **C1417Y** | **38** | **DIV** | **M** | **V** | **1 M** |
| **Present report Pt 4 and Pt 5** | **c.4291dup** | **p.Tyr1431Leufs*27** | **Y1431fs** | **38** | **DIV** | **F** | **V** | **2 M** |

**Supplemental Table 2:** All *NALCN* variants reported in literature associated with IHPRF1 (according to *NALCN* Reference Sequence: NM_052867.4, NP_443099.1). Variants were evaluated for exon, location (Domain: DI, DII, DIII, DIV or extradomain), type of mutation (N: nonsense, F: frameshift, L: splicing, M: missense, S: synonimus) and class according to ACMG guidelines (III: VoUS, IV: Likely Pathogenetic, V: Pathogenetic). Abbreviations: F: female, M: male, NA: not available
