# Supplementary material for: Widening the infantile hypotonia with psychomotor retardation and characteristic Facies-1 Syndrome’s clinical and molecular spectrum through NALCN in-silico structural analysis
Source: Front Genet. 2024 Dec 11;15:1477940. doi: 10.3389/fgene.2024.1477940 (PMC11668739; doi:10.3389/fgene.2024.1477940)
Supplement: Supplementary file 2 [file Image1.pdf]

Supplemental Figures

Supplemental Figure 1

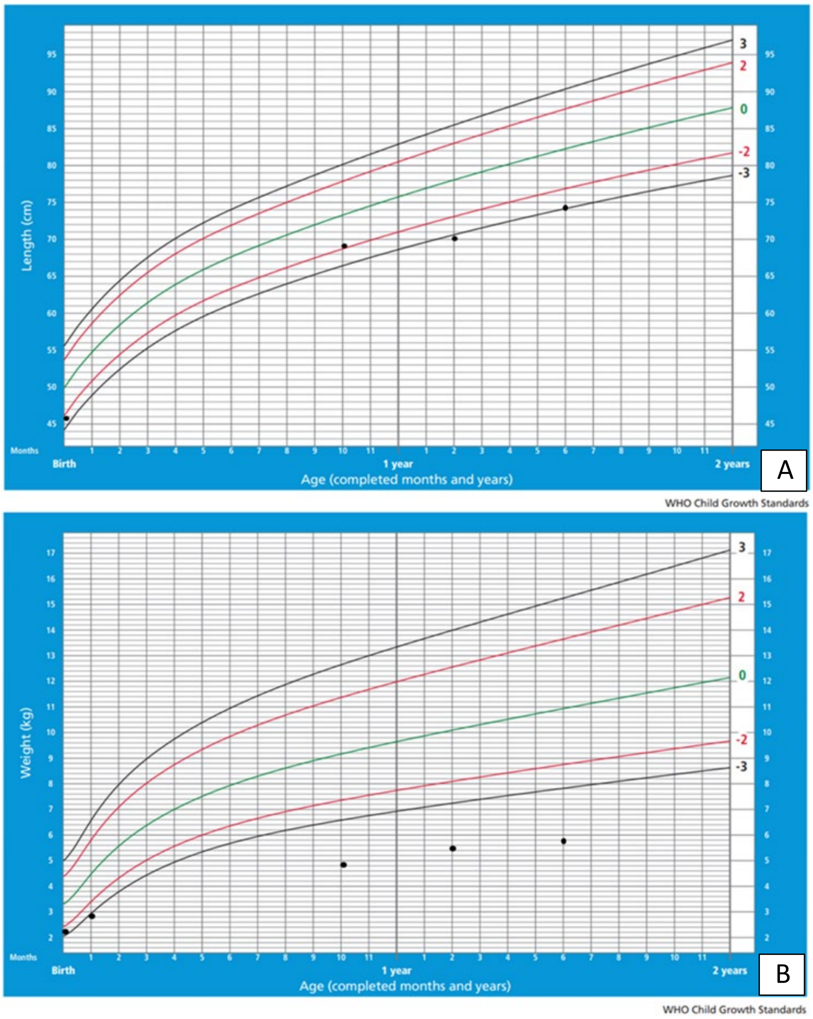

Supplemental Figure 1: Patient growth charts. Panel 1A: Length chart showing linear growth between -2 and -3 SD. Panel 1B: Weight chart showing progressive failure to thrive with a curve below -3 SD

Supplemental Figure 2

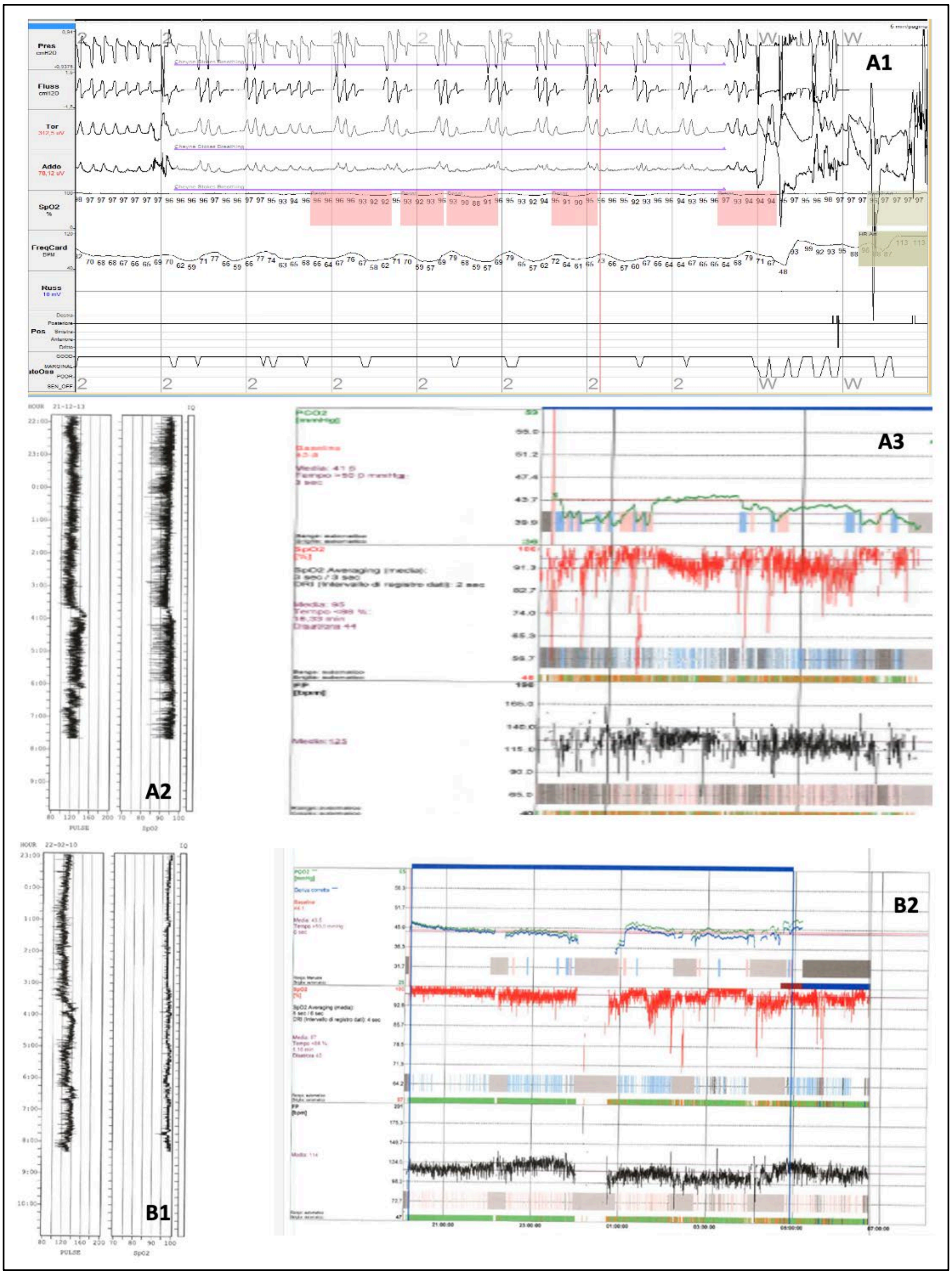

***Supplemental Figure 2*:** Cardiorespiratory study. Panel A1: Without supplemental oxygen: cardiorespiratory study without oxygen supplemental detected periodic breathing at a higher percentage than normal for age: 79.8%. Panels A2 and A3: Without supplemental oxygen: periodic breathing was associated with severe desaturations. Pulse oxymetry reported ODI desaturation index (desaturation numberper hour) was 86.7 and oxygen saturation time < 90% was 7.2% of all time of registration. Values of transcutaneous carbon dioxide were normal, always < 50 mmHg. Periodic breathing percentage at polygraphy has reduced to 66%. Panels: B1 and B2: With supplemental oxygen: pulse oximetry during low flow oxygen therapy 0.5 lt/min detected normal values, ODI 1.5, with saturation constantly > 90%.

Supplemental Figure 3

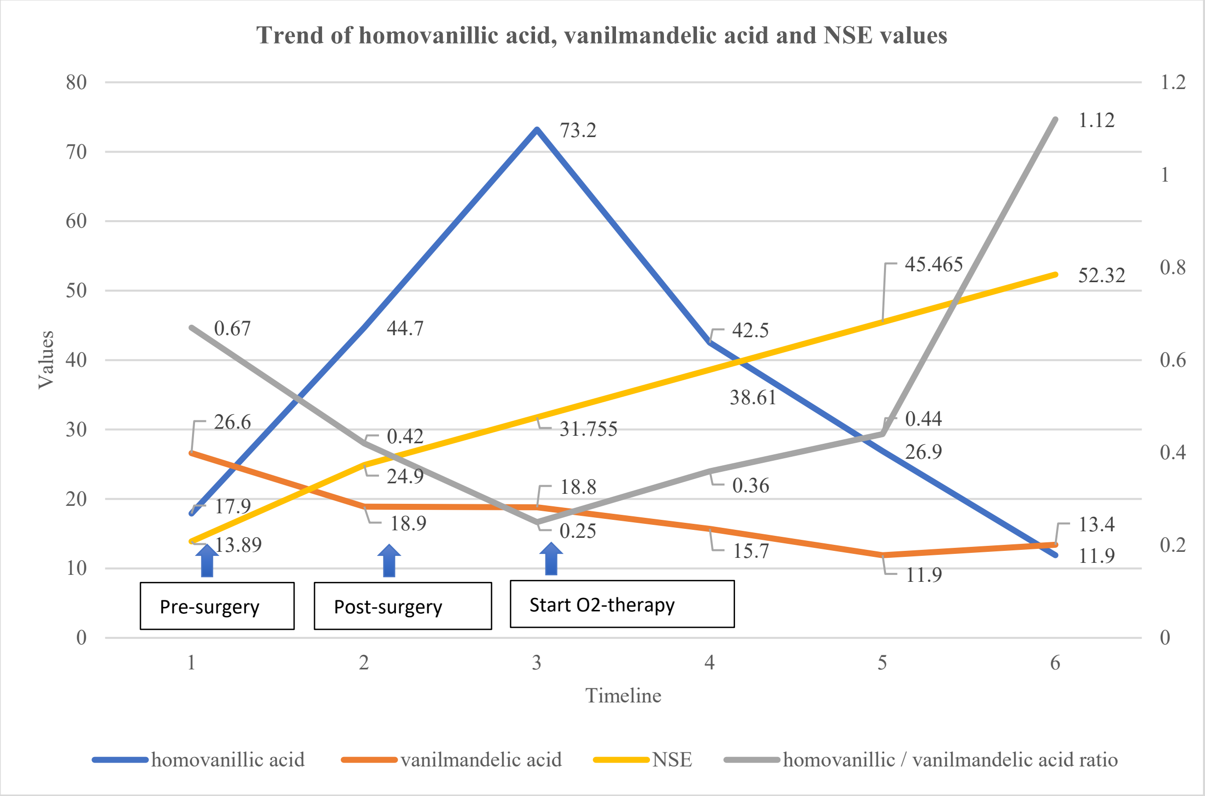

***Supplemental Figure 3*:** Trend of homovanillic acid, vanilmandelic acid, homovanillic/vanilmandelic acid ratio and NSE values over the follow-up. Abbreviation: NSE: Neuron Specific Enolase.
